# Positive and Negative Syndrome Scale (PANSS) predictors of hospitalization during home treatment on 1045 patients with schizophrenia in acute crisis

**DOI:** 10.1192/j.eurpsy.2023.1332

**Published:** 2023-07-19

**Authors:** R. Talisa, A. Sabaté, D. Córcoles, J. Leon, A. Malagón, A. M. González, M. Bellsolà, P. Samos, M. Á. Jerónimo, L. M. Martín, V. Pérez

**Affiliations:** 1 Xarxa de Salut Mental i Addiccions de la Provincia de Girona, Institut d’Assistència Sanitària (IAS), Parc Hospitalari Martí i Julià, Girona; 2 Institut de Neuropsiquiatria i Addiccions, Parc de Salut Mar; 3 Institut de Neuropsiquiatria i Addiccions, Parc de Salut Mar, Barcelona; 4 Centro de Investigación Biomédica en Red de Salud Mental (CIBERSAM), Madrid; 5 Departamento de Psiquiatría y Medicina Forense, Universitat Autonoma de Barcelona, Barcelona, Spain

## Abstract

**Introduction:**

Several factors related to the risk of requiring psychiatric hospitalization have been described in patients diagnosed with schizophrenia treated with methods other than home treatment. With regard to the symptoms, high global illness severity and positive symptoms of schizophrenia have been most frequently related to the risk of hospitalization in patients with schizophrenia. However, there are no studies describing which clinical factors increase the likelihood of being hospitalized while undergoing home follow-up.

**Objectives:**

To determine which of the clinical factors assessed in the PANSS predict the risk of hospitalization in patients diagnosed with schizophrenia following a home treatment program.

**Methods:**

All patients with schizophrenia who were visited by a home treatment team in Barcelona between January 2017 and December 2021 were included in the study. A comparative, bivariate analysis of each item of the PANSS and of the global results of each category was conducted on those who were hospitalized and those who were not hospitalized. Finally, a logistic regression of each category of the PANSS was done on both groups, controlling for other socio-demographic and clinical factors.

**Results:**

A total of 1045 patients with schizophrenia were evaluated in this study. PANSS positive symptom subscale (PANSS-S), PANSS General Psychopathology, PANSS Excited Component and PANSS Global Score scored higher in patients who were finally hospitalized in a conventional acute treatment unit. Regarding the PANSS negative symptom subscale, no significant differences were found between the two groups.

In patients who required hospitalization, the scores of all the PANSS positive symptom subscale (PANSS-P) items and all items on the PANSS excited component (excitement, tension, hostility, uncooperativeness and poor impulse control) were significantly higher. Some items regarding general psychopathology (Somatic concern, anxiety, guilt feelings, tension, and mannerisms) were also significantly higher in the hospitalization group. Only 3 items—blunted affect, guilt feelings and motor retardation—scored significantly higher in patients who did not require hospitalization. In the logistic regression, only the global score of the PANSS-P reached statistical significance (P = 0.001).

**Conclusions:**

Positive symptoms scored in the PANSS seem to be the most predictive factors of hospitalization regarding clinical symptoms in patients with Schizophrenia following home treatment. Other items regarding exciting symptoms and general psychopathology also showed as relevant regarding the risk of conventional hospitalization in those patients.

**Disclosure of Interest:**

None Declared

